# Acute effects of low-intensity one-legged electrical muscle stimulation on arterial stiffness in experimental and control limbs

**DOI:** 10.1038/s41598-024-56963-w

**Published:** 2024-03-20

**Authors:** Hiroyuki Oda, Mami Fujibayashi, Daisuke Kume, Naoyuki Matsumoto, Masato Nishiwaki

**Affiliations:** 1 Faculty of Health Sciences, Morinomiya University of Medical Sciences, Osaka, Japan; 2 Faculty of Engineering, Osaka Institute of Technology, Osaka, Japan; 3 Faculty of Agriculture, Setsunan University, Osaka, Japan; 4 Faculty of Information Science and Technology, Osaka Institute of Technology, Osaka, Japan; 5 Faculty of Environmental Symbiotic Science, Prefectural University of Kumamoto, Kumamoto, Japan

**Keywords:** Cardiology, Health care, Medical research

## Abstract

The aim of this study was to examine the acute effects of low-intensity one-legged electrical muscle stimulation (EMS) for skeletal muscle on arterial stiffness in EMS and non-EMS legs. Eighteen healthy subjects received two different protocols (Control (CT) and Experimental (ET) trials) in random order on separate days. EMS was applied to the left lower limb at 4 Hz for 20 min at an intensity corresponding to an elevation in pulse rate of approximately 15 beats/min (10.9 ± 5.1% of heart rate reserve). Before and after the experiment, arterial stiffness parameters in the control right leg (CRL) and control left leg (CLL) in CT and non-EMS leg (NEL) and EMS leg (EL) in ET were assessed by pulse wave velocity (baPWV, faPWV) and cardio-ankle vascular index (CAVI). No significant changes in all parameters were observed in either leg in CT. Conversely, in ET, low-intensity, single-leg EMS significantly reduced CAVI, baPWV, and faPWV in the EL, but not in the NEL. Acute, low-intensity single-leg EMS reduces arterial stiffness only in the EL. These data support our idea that physical movement-related regional factors rather than systematic factors are important for inducing acute reductions in arterial stiffness.

## Introduction

Pulse wave velocity (PWV) and cardio-ankle vascular index (CAVI) are widely used as clinical indicators of arterial stiffness^1,2^. Previous studies have indicated that arterial stiffness progressively increases with advancing age even in healthy individuals ^3–5^. Large elastic arteries have the ability of arteries to buffer the pulsation due to blood pressure (BP) and flow, but arterial stiffness with age impairs this ability ^6^. In previous studies, it has been reported that an increase in PWV is associated with an elevated risk of cardiovascular disease and mortality rates ^2,7^. Therefore, increased arterial stiffness is an independent risk factor for future cardiovascular disease (CVD) or mortality ^8^, so preventing arterial stiffness is of paramount importamce regardless of age. Moreover, further scientific evidence of simple and effective methods for preventing arterial stiffness is needed.

It is generally accepted that acute or regular voluntary aerobic exercises such as walking, running, cycling, and swimming are ways of reducing arterial stiffness ^9–14^. Passive muscle contractions induced by electrical muscle stimulation (EMS) have recently been gaining attention as a means of reducing arterial stiffness. Indeed, previous studies have shown that acute or long-term EMS intervention reduces arterial stiffness ^15,16^. Regardless of voluntary or passive interventions, these results mean that exercise stimuli in the form of physical movements may represent a key factor in reducing arterial stiffness.

Low-intensity voluntary one-legged physical exercise (resistance exercise, cycling) has been shown to acutely reduce arterial stiffness in the exercised limb, but not in the non-exercised limb ^17,18^. That is, the effects are not transferred from exercised to non-exercised limbs, so regional factors such as exercise stimuli are mainly thought to be important in modulating arterial stiffness, particularly in peripheral arteries. Our recent study also indicated that even low-intensity EMS (approximately 50 mA) can reduce arterial stiffness and that the reduction in arterial stiffness is not uniform among segments ^19^. In the case of EMS of the lower limbs, reductions in arterial stiffness were found in the lower limbs, which received EMS, but not in the upper limbs and trunk, which did not. Since the heart rate during low-intensity EMS does not get as high as in general low-intensity voluntary aerobic exercise, the marked alterations to the systemic circulation seen in low-intensity voluntary aerobic exercise do not occur with low-intensity EMS. When comparing the EMS side to non-EMS side, it is unlikely that the effects transfer with low-intensity EMS. However, the impact of unilateral leg stimulation on arterial stiffness of the non-EMS side remains unclear. Conducting this study has the potential to contribute to the elucidation of the reduction mechanism in arterial stiffness induced by EMS. In addition, these accumulated findings suggest that physical movement-related regional factors can be as important as exercise stimuli to induce acute reductions in arterial stiffness. Accordingly, we speculated that low-intensity, one-legged EMS of skeletal muscle might induce physical movement-related regional factors, thereby reducing arterial stiffness only in the limb receiving EMS. Understanding the modulation of arterial stiffness due to acute low-intensity is important for developing an effective exercise program to reduce arterial stiffness. To the best of our knowledge, however, no data are available regarding one-legged EMS and arterial stiffness.

Therefore, we hypothesized that low-intensity, one-legged EMS of skeletal muscle would induce reductions in arterial stiffness only in the limb to which EMS was applied. The present study thus examined the hypothesis using an acute design as a pilot study into the physiological mechanisms underlying the effects of EMS on arterial stiffness.

## Materials and methods

### Subjects

Participants in this study were 18 male Japanese college students at Osaka Institute of Technology. The students cohort at the institution consists mainly of males, and there were no responses from female students to local advertisement or referrals. Therefore, only males participated in this study. All participants were not obese (BMI < 30 kg/m^2^) and did not have hypertension (systolic BP < 140 mmHg, diastolic BP < 90 mmHg). In addition, none of the participants was smokers and were not taking any medications or supplements at the time of enrollment. Participants had not participated in any exercise, such as club, team, or extracurricular sporting activities for at least the previous 2 years. Table 1 shows baseline characteristic participants in this study. The purpose, procedures, potential benefits, and risks of the study were explained to all participants and written informed consent obtained before experiment from all participants. This study was approved by Human Ethics Committee at Osaka Institute of Technology (approval no. 2016-12). All methods were conducted in accordance with the guidelines of the Human Ethics Committee at Osaka Institute of Technology and Declaration of Helsinki.

**Table 1:** Baseline characteristic participants in this study.

Number of participants	n = 18
Age (years)	20.4 ± 1.4
Hight (cm)	170.8 ± 3.1
Body mass (kg)	64.1 ± 8.5
Body mass index (kg/m^2^)	22.0 ± 3.1
Body fat (%)	18.7 ± 6.3
Lean body mass (kg)	51.7 ± 3.6
Brachial Systolic blood pressure (mmHg)	122 ± 11
Brachial Diastolic blood pressure (mmHg)	69 ± 6
Glucose (mg/dL)	81 ± 12

### Sample size and experimental procedures

We determined the appropriate and minimum sample sizes before the study by calculations using G*Power version 3.1 (Dusseldorf, Germany). We assumed that CAVI would change by 5–10% based on previous findings ^19–21^. Experiments were calculated to require a minimum of 18 participants to detect significant differences with 80% power and a two-tailed α of 5% in repeated-measures two-way analysis of variance (ANOVA) interactions.

Experimental procedures in this study were based on our previous study ^19^. Briefly, all participants were assigned in random sequence (control trial (CT) and experimental trial (ET)) to undergo one trial a day on two separate days, at an interval of approximately one week (mean time between test sessions, 5 ± 1 days). Both trials were conducted in a quiet, air-conditioned room at 24–25 °C. To avoid potential diurnal variations, the two experiments for each participant were performed at the same time of day (range of start time, 10:00–17:00), at least 4 h after a light meal. All participants abstained from vigorous exercise for at least 24 h before testing and from caffeine and food for 4 h before testing. In addition, participants were instructed to eat their regular meals (breakfast, lunch, and dinner) the day before each experiment, and similar standard contents and mealtimes without irregularities within and between participants were confirmed from checklist questionnaires.

**Figure 1:**
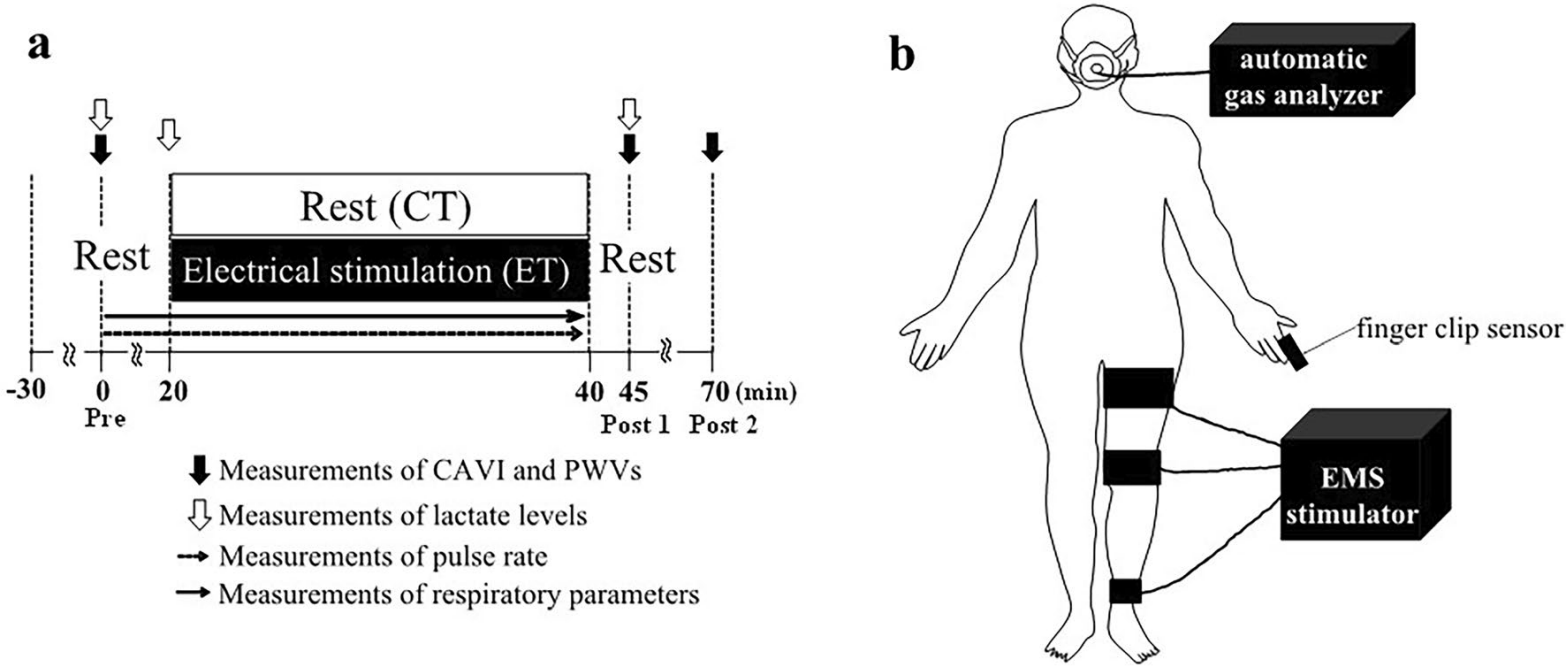
Time course of the experimental protocol (**a**) and experimental setup (**b**). CT, control trial; ET, electrical stimulation trial; CAVI, cardio-ankle vascular index; PWV, pulse wave velocity. Pre, Pre-experiment; Post 1, after experiment (5 min); Post 2, after experiment (30 min); Black and white arrows show time point of measure (black arrow: CAVI, PWVs, white arrow: lactate level). All participants performed each condition in random order on different days.

In both trials, all participants arrived at the laboratory and rested for at least 30 min, then PWVs, hemodynamic parameters, and blood lactate concentrations were assessed in the supine position to establish pre-trial baselines (Rest phase). In the ET, after sitting for 20 min, participants underwent 20 min of EMS of the left lower limb using an electrical stimulator (B-SES SL-1; Homer Ion Japan, Tokyo, Japan) in a sitting position on a bed, as previously described ^19^. After the EMS (recovery phase), measurements of PWVs, hemodynamic parameters, and blood lactate concentration were taken while in a supine position (Fig. 1a). Three rubber stimulation surface electrodes were placed on the left thigh proximally (under the hip), distally (above the knee) and on the ankle (Fig. 1b). This stimulation surface electrode is attached entire rubber band. Thus, range of stimulation is throughout the lower muscle. Stimulator output was set in metabolism mode (4 Hz) because high-frequency electrical stimulation was considered likely to induce tetanic muscle activity and subsequent muscle fatigue ^22^. In addition, all participants did not feel uncomfortable and painful during EMS trial in this study. In this study, stimulation intensity was manually adjusted individually for each patients to create an elevation of approximately 15 beats/min (15 ± 7 beats/min, corresponding to 10.9 ± 5.1% of the heart rate reserve) in pulse rate (PR) ^19^. Indeed, in this study the adjustment of intensity during EMS was very small (proximally thigh: 3 ± 2 mA, distally thigh: 2 ± 2 mA). As electrocardiographic signals and heart rate could not be appropriately recorded during EMS due to interference from the EMS. Therefore, in this study, we monitored PR using a finger clip sensor (SAT-2200; Nihon Kohden, Tokyo, Japan) set at the relative intensity. On the other hand, in the CT rested on the same bed as ET for 40 min. Additionally, PR and respiratory gas parameters were continuously monitored as in ET. After the experimental phase in both trials, each participant rested in a comfortable chair for another 30 min while biometric measurements were repeated at 5 min (Post 1) and 30 min (Post 2).

### Assessment of each parameter

During each trial (i.e., in the experimental phase), minute oxygen uptake (${\dot{\text{V}}}$O_2_), carbon dioxide output (${\dot{\text{V}}}$CO_2_), expired ventilation (${\dot{\text{V}}}$E), and respiratory exchange ratio (RER) were determined every 15 s using an automatic gas analyzer with a mixing chamber (AR-10; Arco System, Chiba, Japan) as a previous study^19^. Each respiratory gas parameter was then automatically calculated using analysis software (AT Windows; Minato Medical Science, Osaka, Japan) and averaged every 5 min. In addition, approximate metabolic equivalents (METs) were calculated by dividing ${\dot{\text{V}}}$O_2_ at 30 min by ${\dot{\text{V}}}$O_2_ at baseline. The day-to-day coefficient of variation (CV; a measure of reproducibility) of ${\dot{\text{V}}}$O_2_ measurement during the same exercise was 5.3 ± 1.5%, as determined in eight individuals on two separate days ^23^. The lactate level before (baseline), 20 min, 45 min (Post 1), and 70 min (Post 2), were measured using a lactate analyzer (Lactate Pro 2; Arklay, Kyoto, Japan). A blood sample was taken from the index finger on the left hand.

BP and PWVs were measured with the participant supine using a semi-automated device (VS-1500AE/AN; Fukuda Denshi, Tokyo, Japan) as assessment parameters for each trial before and 5 and 30 min after each trial, as described previously ^19,21,24^. These values were measured for the control left leg (CLL) and control right leg (CRL) in CT and for the non-EMS leg (NEL) and EMS leg (EL) in ET. Cuffs to measure BP and PWV were wrapped around both upper arms and ankles, then CAVI and brachial-ankle PWV (baPWV) were used as indices of arterial stiffness. CAVI was automatically calculated from five to six pulse-wave signals and arm BP. The baPWV was calculated by dividing the distance between brachial and ankle by the transit time as described. The transit time were determined from the time delay between the brachial and ankle waveforms. Pass length between the brachial and ankle were calculated from heigh of the participant ^25–27^. Furthermore, carotid-femoral PWV (cfPWV) was measured as an index of central arterial stiffness using the same device in another measurement mode. Carotid and femoral arterial pressure waveforms were recorded using amorphous pulse wave sensors (TY-501A; Fukuda Denshi) attached to the carotid and femoral arteries, and values were automatically calculated as the distance between the carotid and femoral artery sites divided by transit time ^19,21^. In this study, femoral-ankle PWV (faPWV) was also calculated as the distance between femoral and ankle artery sites divided by femoral–ankle transit time. The CVs for CAVI, baPWV, and cfPWV measurements on two separate days (reproducibility) were 3.6 ± 0.6%, 2.7 ± 0.3%, and 7.5 ± 1.2%, respectively ^25,28^.

### Statistical analysis

Results are presented as mean ± standard deviation (SD). The normality of data was first assessed using the Shapiro-Wilks test. Changes in each parameter were analyzed by two-way (conditions × time points) repeated-measures ANOVA. When the F-value was significant, Bonferroni correction was applied for post-hoc multiple comparisons. All data were statistically analyzed using Sigma Stat version 2.03 (Systat Software Inc., San Jose, CA, USA). The significant level was P < 0.05 for all data.

## Results

### Changes in physiological parameters during CT and ET

**Figure 2:**
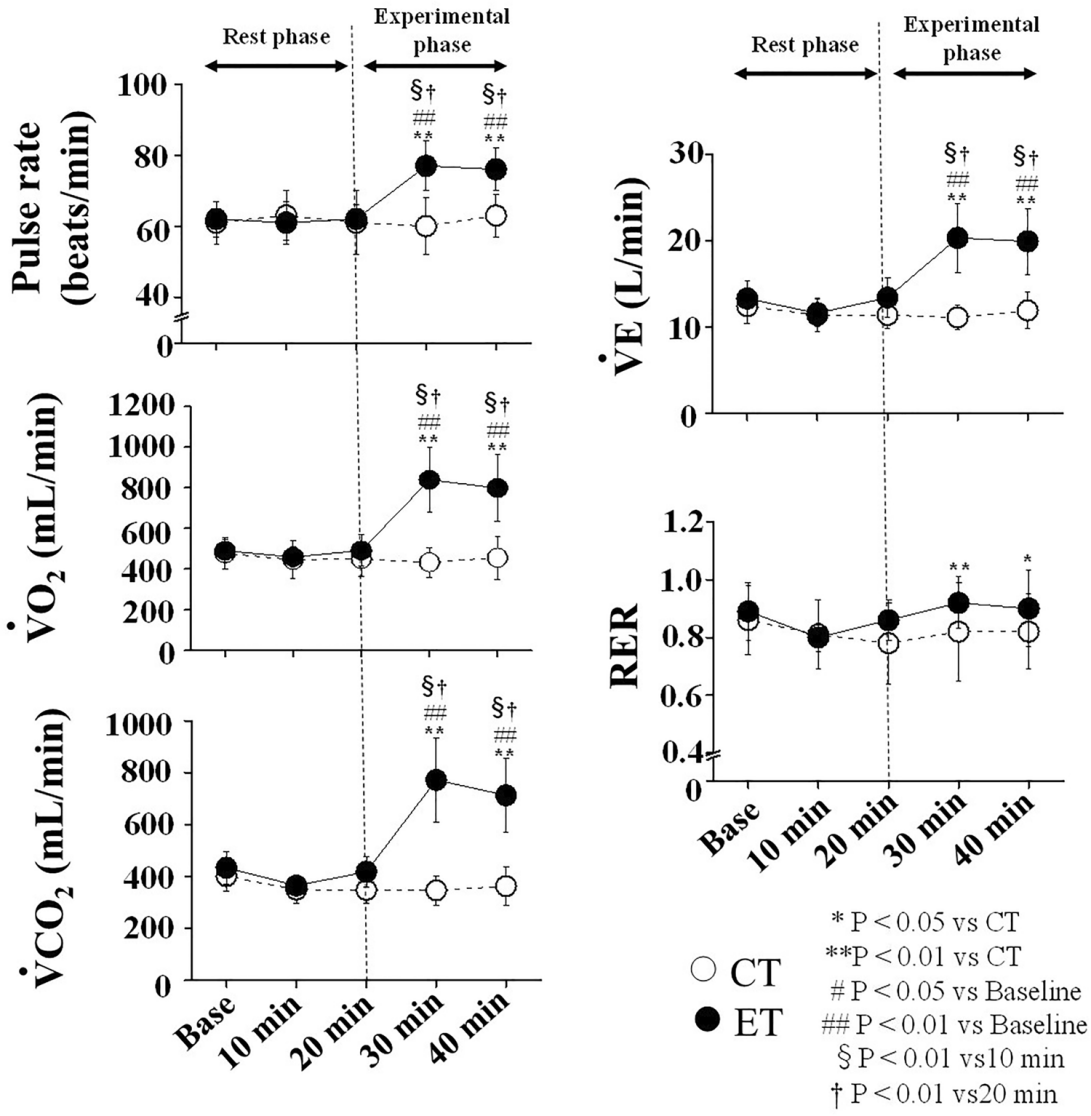
Temporal changes in pulse rate and respiratory parameters. Open circles (○) and filled circles (●) show CT (Control trial) and ET (Electrical stimulation trial), respectively. The vertical dash line is the starting point of EMS. ${\dot{\text{V}}}$O2, oxygen uptake; ${\dot{\text{V}}}$CO2, carbon dioxide output; ${\dot{\text{V}}}$E, minute expired ventilation; RER, respiratory exchange ratio; * P < 0.05 vs CT; ** P < 0.01 vs CT; # P < 0.05 vs Baseline; ## P < 0.01 vs Baseline; §P < 0.01 vs 10 min; †P < 0.01 vs 20 min. Data are given as a mean ± SD.

Two-way repeated-measures ANOVA revealed significant interactions in PR and respiratory gas parameters, respectively (PR: P < 0.001, ${\dot{\text{V}}}$O_2_: P < 0.001, ${\dot{\text{V}}}$CO_2_: P < 0.001, VE: P < 0.001, RER: P = 0.01; Table 2). No significant differences in these baseline parameters were observed between the two trials, and parameters of the CT did not change significantly throughout the study period. Conversely, PR in the ET increased significantly from baseline during EMS (15 ± 7 beats/min) and was significantly higher than in CT (17 ± 12 beats/min). During EMS, each respiratory gas parameter increased significantly from baseline and values were higher than those in CT, similar to PR (Fig. 2, Table 2). Circulating lactate levels in the ET were significantly increased only at Post 1 (CT: 1.1 ± 0.3 mmol/L, ET: 2.1 ± 0.6 mmol/L; P < 0.001).

**Table 2:** Temporal change in pulse rate and respiratory parameters.

	Phase	Baseline			10 min			20 min			30 min			40 min			Interaction	
		Rest			Rest			Rest			Experimental			Experimental				
Pulse rate (beats/min)	CT	61 ± 6			63 ± 7			61 ± 9			60 ± 8			63 ± 6			F = 20.259	P < 0.001
	ET	62 ± 5			61 ± 6			62 ± 4			77 ± 7**##§†			76 ± 6**##§†				
${\dot{\text{V}}}$O_2_ (mL/min)	CT	477 ± 76			445 ± 94			449 ± 86			432 ± 73			455 ± 106			F = 73.286	P < 0.001
	ET	489 ± 56			459 ± 52			491 ± 77*			839 ± 162**##§†			798 ± 164**##§†				
${\dot{\text{V}}}$CO_2_ (mL/min)	CT	402 ± 58			349 ± 53			348 ± 50			347 ± 57			363 ± 74			F = 75.791	P < 0.001
	ET	434 ± 63			365 ± 35			418 ± 57			772 ± 161**##§†			713 ± 144**##§†				
${\dot{\text{V}}}$E (L/min)	CT	12.43 ± 2.05			11.39 ± 1.90			11.40 ± 1.63			11.13 ± 1.37			11.87 ± 2.12			F = 61.068	P < 0.001
	ET	13.26 ± 2.05			11.55 ± 1.61#			13.35 ± 2.30**			20.27 ± 4.01**##§†			19.95 ± 3.85**##§†				
RER	CT	0.86 ± 0.12			0.81 ± 0.12			0.78 ± 0.14			0.82 ± 0.17			0.82 ± 0.13			F = 3.600	P = 0.01
	ET	0.89 ± 0.10			0.80 ± 0.05			0.86 ± 0.07*			0.92 ± 0.09**			0.90 ± 0.13*§†				

Data are expressed as mean ± SD. CT, control trial; ET, experimental trial. *P < 0.05 vs CT; **P < 0.01 vs CT; #P < 0.05 vs Baseline; ## P < 0.01 vs Baseline; § P < 0.01 vs 10 min;† P < 0.01 vs 20 min. ${\dot{\text{V}}}$O_2_, oxygen uptake; ${\dot{\text{V}}}$CO_2_, carbon dioxide output; ${\dot{\text{V}}}$E, minute expired ventilation; RER, respiratory exchange ratio.

### Effects of EMS on arterial stiffness and hemodynamic parameters

**Figure 3:**
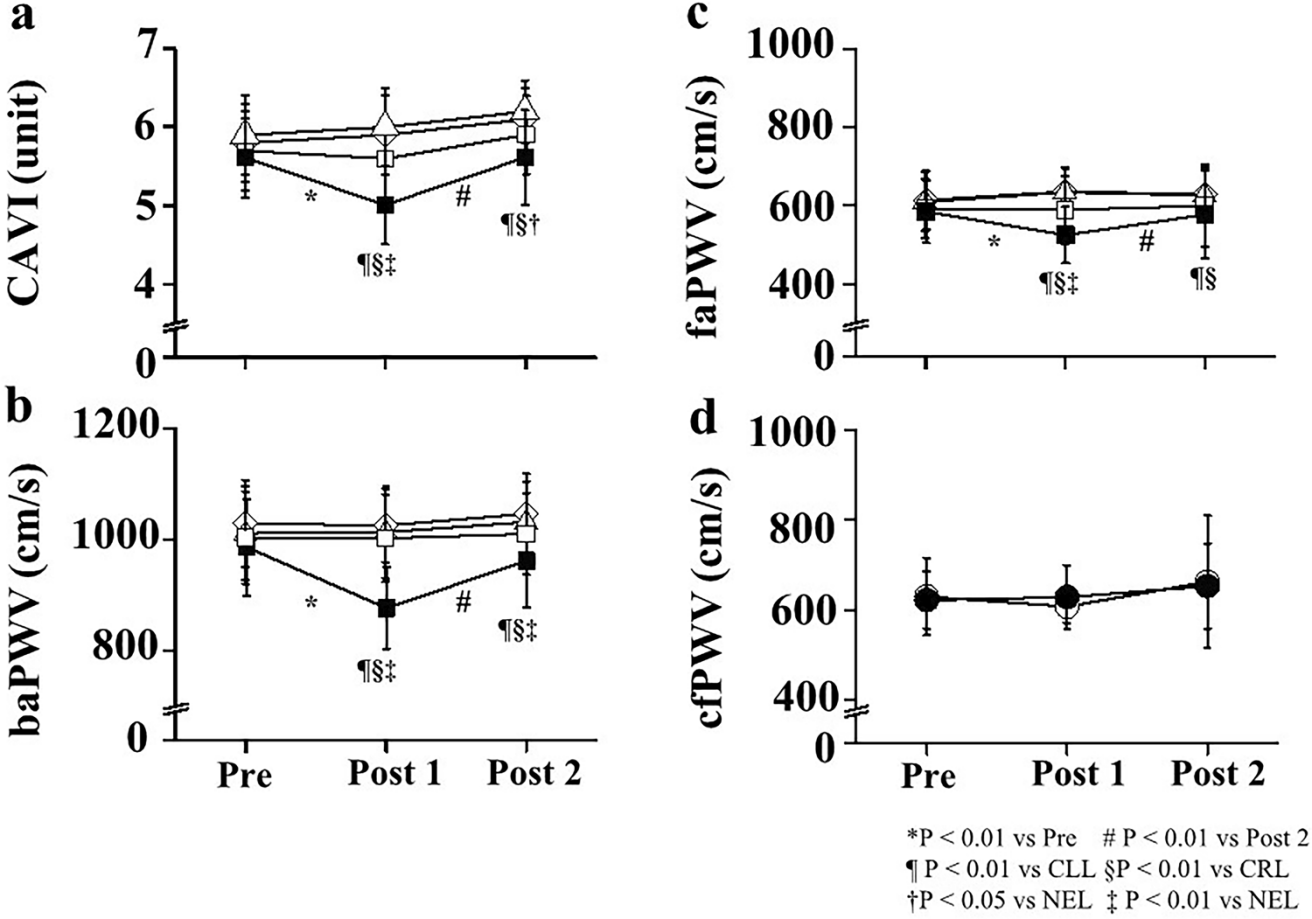
Temporal change in arterial stiffness parameters. Changes in values for CAVI (**a**), baPWV (**b**), faPWV (**c**), and cfPWV (**d**). CLL, control left leg; CRL, control right leg; NEL, non-electrical stimulation leg; EL, electrical stimulation leg; CT, control trial; ET, electrical stimulation trial; CAVI, cardio-ankle vascular index; baPWV, brachial-ankle pulse wave velocity; faPWV, femoral-ankle PWV; cfPWV, carotid-femoral pulse wave velocity; Pre, pre-experiment; Post 1, 5 min post-experiment; Post 2, 30 min post-experiment. Open rhombus (◇), control right leg; open triangle (△), control left leg; open square (□), non-electrical stimulation leg; filled square (■), electrical stimulation leg; open circles (○), control trial; filled circles (●), electrical stimulation trial. * P < 0.01 vs Pre; # P < 0.01 vs Post 2; ¶ P < 0.01 vs CLL; § P < 0.01 vs CRL; † P < 0.05 vs NEL; ‡ P < 0.01 vs NEL. Data are given as a mean ± SD.

Two-way repeated-measures ANOVA revealed significant interactions in CAVI, baPWV, and faPWV (P < 0.001 respectively), but not in cfPWV (P = 0.267) (Table 3). CAVI and PWVs at Pre did not differ significantly between trials, and CAVI and PWVs in the CRL, CLL, and NEL did not change significantly throughout the study period. On the other hand, CAVI, baPWV, and faPWV in the EL were all significantly reduced at Post 1 (P < 0.01 respectively). CAVI, baPWV, and faPWV values at Post 1 were also significantly lower in the EL than in the CRL, CLL, and NEL (P < 0.01 respectively). Moreover, CAVI, baPWV, and faPWV values at Post 2 were significantly lower in the EL than in the CRL and CLL. However, cfPWV did not change significantly in either CT or ET (Fig. 3; Table 3).

**Table 3:** Temporal change in arterial stiffness parameters.

			Pre			Post 1			Post 2			Interaction	
CAVI (unit)	CT	CRL	5.9 ± 0.5			6.0 ± 0.5			6.2 ± 0.4			F = 9.464	P < 0.001
		CLL	5.8 ± 0.5			5.9 ± 0.5			6.1 ± 0.4				
	ET	NEL	5.7 ± 0.5			5.6 ± 0.5			5.9 ± 0.5				
		EL	5.6 ± 0.5			5.0 ± 0.5*#¶§‡			5.6 ± 0.6¶§†				
ba PWV (cm/s)	CT	CRL	1029 ± 78			1025 ± 66			1046 ± 72			F = 18.048	P < 0.001
		CLL	1011 ± 84			1013 ± 83			1031 ± 73				
	ET	NEL	1003 ± 83			1002 ± 79‡			1010 ± 74				
		EL	986 ± 87			877 ± 73*#¶§‡			962 ± 84¶§‡				
fa PWV (cm/s)	CT	CRL	614 ± 75			636 ± 57			629 ± 69			F = 5.071	P < 0.001
		CLL	609 ± 76			634 ± 61			626 ± 69				
	ET	NEL	592 ± 77			589 ± 86			599 ± 105				
		EL	584 ± 80			525 ± 73*#¶§‡			577 ± 113¶§‡				
cf PWV (cm/s)	CT		629 ± 86			607 ± 36			663 ± 147			F = 1.315	P = 0.267
	ET		622 ± 64			628 ± 71			653 ± 95				

Data are expressed as mean ± SD. CT, control trial; ET, experimental trial; CRL, control right leg; CLL, control left leg; NEL, non-electrical stimulation leg; EL, electrical stimulation leg. * P < 0.01 vs Pre; # P < 0.01 vs Post 2; ¶ P < 0.01 vs CLL; § P < 0.01 vs CRL; † P < 0.05 vs NEL; ‡ P < 0.01 vs NEL.

Two-way repeated-measures ANOVA revealed no significant interactions in arm BPs (systolic P = 0.116, diastolic P = 0.156, mean P = 0.075; Table 4). Arm BPs at Pre did not differ significantly between either trial. Diastolic BPs at Post 1 and Post 2 were slightly but significantly increased compared with both Pre and Post 1 in the CRL and CLL in CT, but not in the NEL or EL in ET. Conversely, two-way repeated-measures ANOVA revealed significant interactions in ankle BPs (systolic P < 0.001, diastolic P < 0.001, mean P < 0.001; Table 4). Ankle BP at Pre did not differ significantly between any trials. However, systolic, diastolic, and mean BPs at Post 1 were significantly lower in the EL than in the CRL, CLL, or NEL (P < 0.01 vs CRL, vs CLL, vs NEL respectively; Table 4). Diastolic BP at Post 2 was also significantly lower in the EL than in the CRL (P < 0.05; Table 4). Systolic, diastolic and mean BPs at Post 1 were significantly reduced compared with Pre and Post 2 in EL (vs Pre: systolic P < 0.01, diastolic P < 0.01, mean P < 0.01; vs Post 2: systolic P < 0.01, diastolic P < 0.01, mean P < 0.01; Table 4).

**Table 4:** Temporal changes in arm and ankle blood pressure.

				Pre			Post 1			Post 2			Interaction	
Systolic blood pressure (mmHg)	Arm	CT	CRL	125 ± 13			125 ± 12			122 ± 11			F = 1.755	P = 0.116
			CLL	119 ± 11			122 ± 10			121 ± 11				
		ET	NEL	125 ± 14			125 ± 13			124 ± 12				
			EL	123 ± 8			122 ± 10			120 ± 8				
	Ankle	CT	CRL	132 ± 11			136 ± 11			137 ± 10*			F = 6.146	P < 0.001
			CLL	132 ± 11			134 ± 6			136 ± 11*				
		ET	NEL	133 ± 13			137 ± 13			138 ± 10*				
			EL	136 ± 11			127 ± 10**##¶§§†			133 ± 10				
Diastolic blood pressure (mmHg)	Arm	CT	CRL	69 ± 6			71 ± 7*#			74 ± 6			F = 1.594	P = 0.156
			CLL	69 ± 7			69 ± 5			73 ± 6**				
		ET	NEL	71 ± 9			72 ± 8			72 ± 9				
			EL	71 ± 7			70 ± 6			73 ± 9				
	Ankle	CT	CRL	63 ± 5			67 ± 7**			70 ± 6**			F = 10.528	P < 0.001
			CLL	65 ± 4			67 ± 5			69 ± 4*				
		ET	NEL	66 ± 6			67 ± 6			67 ± 9				
			EL	66 ± 5			57 ± 6**##¶§§†			65 ± 7§				
Mean blood pressure (mmHg)	Arm	CT	CRL	89 ± 7			91 ± 8			91 ± 7			F = 1.984	P = 0.075
			CLL	88 ± 7			88 ± 6			91 ± 7				
		ET	NEL	91 ± 10			92 ± 9			91 ± 9				
			EL	91 ± 7			89 ± 6			91 ± 8				
	Ankle	CT	CRL	88 ± 8			91 ± 8			92 ± 9			F = 7.870	P < 0.001
			CLL	88 ± 6			91 ± 7			93 ± 6**				
		ET	NEL	88 ± 7			89 ± 9			92 ± 8				
			EL	89 ± 8			80 ± 6**##¶§§†			88 ± 9				

Data are expressed as mean ± SD. CT, control trial; ET, experimental trial; CRL, control right leg; CLL, control left leg; NEL, non-electrical stimulation leg; EL, electrical stimulation leg. * P < 0.01 vs Pre; # P < 0.01 vs Post 2; ¶ P < 0.01 vs CLL; § P < 0.05 vs CRL; §§ P < 0.01 vs CRL; † P < 0.05 vs NEL; †P < 0.01 vs NEL.

## Discussion

The salient findings from this investigation were that acute, low-intensity EMS to the left lower limb significantly reduced CAVI, baPWV, and faPWV in the EL, but not baPWV and faPWV in the NEL, with no accompanying changes in cfPWV. These findings suggest that acute, low-intensity, one-legged EMS reduces arterial stiffness only in the limb receiving EMS and supports the notion that physical movement-related regional factors are important in acutely reducing arterial stiffness.

In the present study, the intensity of EMS was set at 47 ± 22 mA (proximally thigh), 32 ± 5 mA (distally thigh). Moreover, there was an increase of 15 ± 7 beats/min in PR compared to the baseline value, and the mean PR of 77 ± 6 beats/min (10.9 ± 5.1% of HRR) is classified as low-intensity exercise stimulus ^29^. Respiratory gas parameters in the ET also significantly increased during EMS and the intensity of energy metabolism corresponded to approximately 2 METs (1.7 ± 0.3), the same level as standing ^30^. Although lactate levels increased slightly after EMS, the mean value of 2.1 ± 0.6 mmol/L (Post 1) did not reach the threshold for onset of blood lactate accumulation (4 mmol/L) ^31,32^. Therefore, in terms of physiological indicators during EMS, the intensity of our one-legged EMS was the same as or lower than walking, confirming that our EMS was low-intensity.

Although a previous study showed that acute, low-intensity EMS reduces arterial stiffness in sites local to the target site for EMS ^19^, our new findings indicate reductions in baPWV and faPWV only in the EL following acute, low-intensity, one-legged EMS. A previous study by Sugawara et al. ^18^ has demonstrated that following acute, low-intensity, one-legged aerobic exercise, there was an approximately 8% reduction in faPWV of the exercised limb with no change in PWV of the non-exercised limb. Moreover, Heffernan et al.^17^ indicated that acute, one-legged resistance exercise reduced peripheral PWV only in the exercised leg (approximately 13%). We noted that low-intensity, one-legged EMS reduced arterial stiffness in the EL by approximately 10% (baPWV: 10.8 ± 5.9%; faPWV: 9.6 ± 10.4%). The present results are approximately consistent with previous studies of one-legged voluntary exercise ^17,18^. The main influences of one-legged physical movement are also not transferred between exercised and non-exercised limbs, regardless of the voluntary or passive nature of the interventions. Our results thus suggest that physical movement-related regional factors, rather than systemic factors, are important in reducing or improving arterial stiffness.

Alterations in arterial stiffness are generally thought to result from vascular structural changes, vascular functional changes, or a combination of the two ^6^. Because an acute design and approach were applied in this study, vascular structural changes were considered quite unlikely to have been responsible for the reductions in arterial stiffness, due to relatively long term (i.e., at least several weeks) required to effect such changes in anatomical structures ^33^. Conversely, from the perspective of vascular functional changes, vascular smooth muscle tone is mainly known to be affected by α-adrenergic receptor-dependent vasoconstrictor tone, vasoactive substances such as nitric oxide (NO), and so on ^6^. In particular, increased blood flow associated with physical movement has been considered one of the key factors contributing to acute reductions in arterial stiffness as a physical movement-related regional factor. In the present study, passive muscle contractions with one-legged EMS also significantly increased PR and ${\dot{\text{V}}}$O_2_ despite the low intensity of EMS. The results suggest that one-legged EMS increased oxygen demands in the EMS-performed limb, thereby bringing about an increase in blood flow with physical movements in the EL. Supporting these circulatory changes, ankle BPs (systolic, diastolic, and mean BPs) were significantly reduced in EL. These data suggest that low-intensity EMS can thus reduce peripheral vascular resistance, resulting in increased blood inflow. In addition, regional physical movements might increase shear stress, triggering NO release. Repetitive increases in blood flow or shear stress caused by one-legged EMS might thus increase NO levels in the vascular endothelium, in turn reducing arterial stiffness. Indeed, previous studies have demonstrated that passive muscle contractions due to EMS cause increases in shear stress and blood flow ^34,35^. Furthermore, previous in vivo studies have shown that NO reduces arterial stiffness ^36^ and that endothelial NO synthase is associated with increased production of NO following EMS ^37^. However, we did not obtain direct regional evidence in vivo to support this notion and further investigations are required.

In generally, central arterial stiffness has been identified as an independent risk factor for future CVD ^1,38^. However, in recent previous study, peripheral arterial stiffness (baPWV, faPWV, ABI; ankle-brachial index) has also been reported as an independent risk factor for CVD^39–42^. In addition, Vlachopoulos et al.^2,7^ demonstrated that each 1 m/s increase in PWV corresponding to a > 10% increase in the risk of cardiovascular events or mortality, so reduction in arterial stiffness are of paramount importance. Interestingly, in this study, low-intensity EMS reduced arterial stiffness to approximately the level of reduction seen with one-legged voluntary exercise in the previous study ^17,18^. Nevertheless, the EMS of physical intensity as assessed by ${\dot{\text{V}}}$O_2_ was equivalent to standing. Moreover, it resulted in a decrease of 1 m/s (109.2 ± 63.7 cm/s) in baPWV and 0.6 m/s (59.6 ± 60.1 cm/s) in faPWV. Therefore, the reduction induced by low-intensity EMS may offer the potential to provide a novel exercise program for preventing CVD. In the future, it will be necessary to explore practical applications in a clinical setting, including consideration of the using time and optimal intensity.

Finally, several important limitations need to be considered for this study. First, we calculated appropriate sample sizes before the study, but the cohort of participants was homogeneous, comprising healthy young male individuals. In general, large elastic arteries progressively stiffen with age ^28,43^. Additional interventions for elderly subjects and individuals with high blood pressure may thus reveal important insights into the alterations and adaptations of arterial stiffness with EMS. The future investigation studies targeting such populations are needed. Second, in terms of methodological reasons for ultrasound imaging devices, blood flow alterations could not be directly assessed in the experimental limb during EMS. Thus, blood flow could not be confirmed as one of the physical movement-related regional factors in the present study.

In conclusion, the present findings indicate that acute, low-intensity, one-legged EMS reduces arterial stiffness only in the limb receiving EMS. These data strongly support our idea that physical movement-related regional factors rather than systemic factors are important for inducing acute reductions in arterial stiffness.

## Data Availability

The raw data supporting the conclusion of this article will be available by corresponding author, without undue reservation.
